# Alterations in sensorimotor function after ACL reconstruction during active joint position sense testing. A systematic review

**DOI:** 10.1371/journal.pone.0253503

**Published:** 2021-06-25

**Authors:** Aglaja Busch, Angela Blasimann, Frank Mayer, Heiner Baur

**Affiliations:** 1 Sports Medicine & Sports Orthopedics, University Outpatient Clinic, University of Potsdam, Potsdam, Germany; 2 Department of Health Professions, Division of Physiotherapy, Bern University of Applied Sciences, Bern, Switzerland; 3 Department of Rehabilitation Sciences and Physiotherapy, Faculty of Medicine and Health Sciences, University of Antwerp, Antwerp, Belgium; Paracelsus Medizinische Privatuniversitat - Nurnberg, GERMANY

## Abstract

**Background:**

The anterior cruciate ligament (ACL) rupture can lead to impaired knee function. Reconstruction decreases the mechanical instability but might not have an impact on sensorimotor alterations.

**Objective:**

Evaluation of the sensorimotor function measured with the active joint position sense (JPS) test in anterior cruciate ligament (ACL) reconstructed patients compared to the contralateral side and a healthy control group.

**Methods:**

The databases MEDLINE, CINAHL, EMBASE, PEDro, Cochrane Library and SPORTDiscus were systematically searched from origin until April 2020. Studies published in English, German, French, Spanish or Italian language were included. Evaluation of the sensorimotor performance was restricted to the active joint position sense test in ACL reconstructed participants or healthy controls. The Preferred Items for Systematic Reviews and Meta-Analyses guidelines were followed. Study quality was evaluated using the Quality Assessment Tool for Observational Cohort and Cross-Sectional Studies. Data was descriptively synthesized.

**Results:**

Ten studies were included after application of the selective criteria. Higher angular deviation, reaching significant difference (p < 0.001) in one study, was shown up to three months after surgery in the affected limb. Six months post-operative significantly less error (p < 0.01) was found in the reconstructed leg compared to the contralateral side and healthy controls. One or more years after ACL reconstruction significant differences were inconsistent along the studies.

**Conclusions:**

Altered sensorimotor function was present after ACL reconstruction. Due to inconsistencies and small magnitudes, clinical relevance might be questionable. JPS testing can be performed in acute injured persons and prospective studies could enhance knowledge of sensorimotor function throughout the rehabilitative processes.

## Introduction

A rupture of the anterior cruciate ligament (ACL) is a severe and long-lasting sports injury. To regain joint stability, a surgical reconstruction is an often chosen treatment [1]. However, reconstruction and rehabilitation are not a guarantee for restored knee function. Impaired knee function is reported, even years after ACL injury, leading to decreased physical activity and poor knee related quality of life [2, 3]. Decreased knee function and higher incidences of subsequent injuries are hypothesised to be the result of changed somatosensory sensation and altered motor outputs [4]. It is assumed that incorporated sensors in the ACL contribute information to the somatosensory perception, which includes the perception of sensory stimuli in the fields of temperature, touch, pain and proprioception [5]. Proprioception in more detail is the afferent information from proprioceptors, including the sensation of motion (kinesthesia), static joint position and force or tension [6]. Contributors to proprioception are mechanoreceptors, muscle spindles and Golgi tendon organs recognizing a stimulus and converting the mechanical energy into electrical energy in form of an action potential. This information, among others, is provided to afferent nerves and sent to the central nervous system [6].

To examine proprioception after ACL rupture and reconstruction in the knee joint the joint position sense (JPS) tests is an often chosen technique [7]. The JPS test can be assessed with active or passive reproduction. During passive reproduction the limb is passively moved to the targeted angle and indicated by the participant when the targeted angle is reached. In contrast, in the active JPS test, the limb is actively moved to the target angle by the participant [8]. Reviews were conducted to summarise the study results of proprioceptive measurements in ACL deficient participants and also after ACL reconstruction [9–11]. However, these reviews are either in need of current literature update or combine different measurement techniques to give a conclusion about proprioceptive performance.

Therefore, the purpose of this systematic review was to determine the sensorimotor function measured with the active JPS test in ACL reconstructed participants compared to the contralateral side and healthy control group. Further, a detailed description of JPS testing protocols will be provided for a comparison and evaluation of used study designs.

## Materials and methods

### Search strategy

This systematic review followed the recommendations of Preferred Reporting Items for Systematic Reviews and Meta-Analyses (PRISMA) [12] and was registered in PROSPERO registry (CRD42020166558).

Electronic databases of MEDLINE, CINAHL, EMBASE, PEDro, Cochrane Library and SPORTDiscus were searched from their origin until April 2020 addressing ACL reconstructed patients performing an active joint positions sense test compared to the contralateral side or to healthy controls. Following search terms/keywords combined with Boolean search operators in title, abstract, MeSH and keywords fields were used: (Anterior cruciate ligament OR ACL) AND (reconstruction OR surgery OR repair) AND (joint position sense test OR JPS test). A pre-selection in the search engine excluding animal studies was implemented if possible. Reference lists of included articles were screened for possible missed articles and completed the search.

### Study selection

Two reviewers (ABu and ABl) independently screened the identified studies for eligibility after excluding duplicates using a web application (Rayyan, Qatar Computing Research Institute, Hamad Bin Khalifa University, Doha, Qatar) [13]. In case of inclusion disagreement of a study, the opinion of a third collaborator (HB) was requested. Inclusion criteria for a full text review were if (1) the study examined patients with a complete ACL rupture (regardless of associate injuries e.g. additional meniscal tears) and surgical reconstruction compared to the contralateral side and/or healthy controls; (2) reporting of the performance of an active knee JPS test; (3) participants of all sexes and between 18–65 years of age. Age limitations were set due to possible effects of aging on joint proprioception [14]. Moreover, publications in English, German, Spanish, French or Italian language were included.

Articles focusing on post-mortem participants or animals were excluded. Further, an exclusion occurred if (1) studies solely using proprioceptive test performances as an outcome after interventional treatment; (2) JPS testing was performed with additional bracing or taping; (3) inclusion of patients with prior history of lower limb musculoskeletal surgeries or suffering from pathologies which could affect the sensorimotor function, such as neurological disorders, knee osteoarthritis, rheumatoid arthritis, patellofemoral pain, jumper´s knee etc. were reported; (4) the identified study was a systematic review, meta-analysis, case series with < 5 participants, book chapter, conference paper or thesis. If no full text of the selected study was available, the corresponding author was contacted; no reply resulted in exclusion of the paper.

### Data extraction

The following information of the included studies were summarized by one (ABu) and independently proved by a second (ABl) reviewer: authors, year of publication, characteristics of participants (age, sex, sports/physical activity, type of surgery, associated injuries, time between surgery and study participation), testing protocol (starting leg, familiarization trial, targeted angles, movement velocity), comparison to affected limb (contralateral limb or healthy control group), primary outcome measures.

### Quality assessment of the included studies

Two independent reviewers (ABu and ABl) assessed the risk of bias and quality of the publications using the Quality Assessment Tool for Observational Cohort and Cross-Sectional Studies [15]. The Quality Assessment Tool [15] gives guidance to determine the internal validity of observational cohort and cross-sectional studies. 14 questions are asked which can be answered by Yes, No or Other (cannot determine, not applicable, not reported). To critically appraise the included studies, the following questions were implemented (1) Was the research question or objective in this paper clearly stated? (2) Was the study population clearly specified and defined? (3) Was the participation rate of eligible persons at least 50%? (4) Were all participants selected or recruited from the same or similar populations (including the same time period)? (5) Was a sample size justification, power description or variance and effect estimates provided? (6) For the analyses in this paper, were the exposure(s) of interest assessed prior to the outcome(s) being measured? (7) Was the timeframe sufficient so that one could reasonably expect to see an association between exposure and outcome if it existed? (8) For exposures that can vary in amount or level, did the study examine different levels of the exposure as related to the outcome (e.g. categories of exposure, or exposure measured as continuous variable)? (9) Were the outcome measures (dependent variables) clearly defined, valid, reliable and implemented consistently across all study participants? (10) Was the exposure(s) assessed more than once over time? (11) Were the outcome measures (dependent variables) clearly defined, valid, reliable and implemented consistently across all study participants? (12) Were the outcome assessors blinded to the exposure status of participants? (13) Was loss to follow-up after baseline 20% or less? (14) Were key potential confounding variables measured and adjusted statistically for their impact on the relationship between exposure(s) and outcome(s)?

Questions (6) to (10) focus on the exposure. However, as this review did not concentrate on interventional studies, the exposure is defined as ACL rupture and reconstruction. Therefore, question (6) to (9) focused on the ACL reconstruction (question (6)) with respect to the description of surgery type (question (8)) and associate injuries (question (9)). As the inclusion criteria only allowed primary ACL tears and reconstruction, re-ruptures and secondary assessments of the exposure were not included and question (10) was always answered with not applicable (NA). The Quality Assessment Tool lacks cut-off values for study quality rating, as studies are subjectively rated as good, fair or poor based on the bias assessed in the previously described questions [15].

## Results

**Fig 1:**
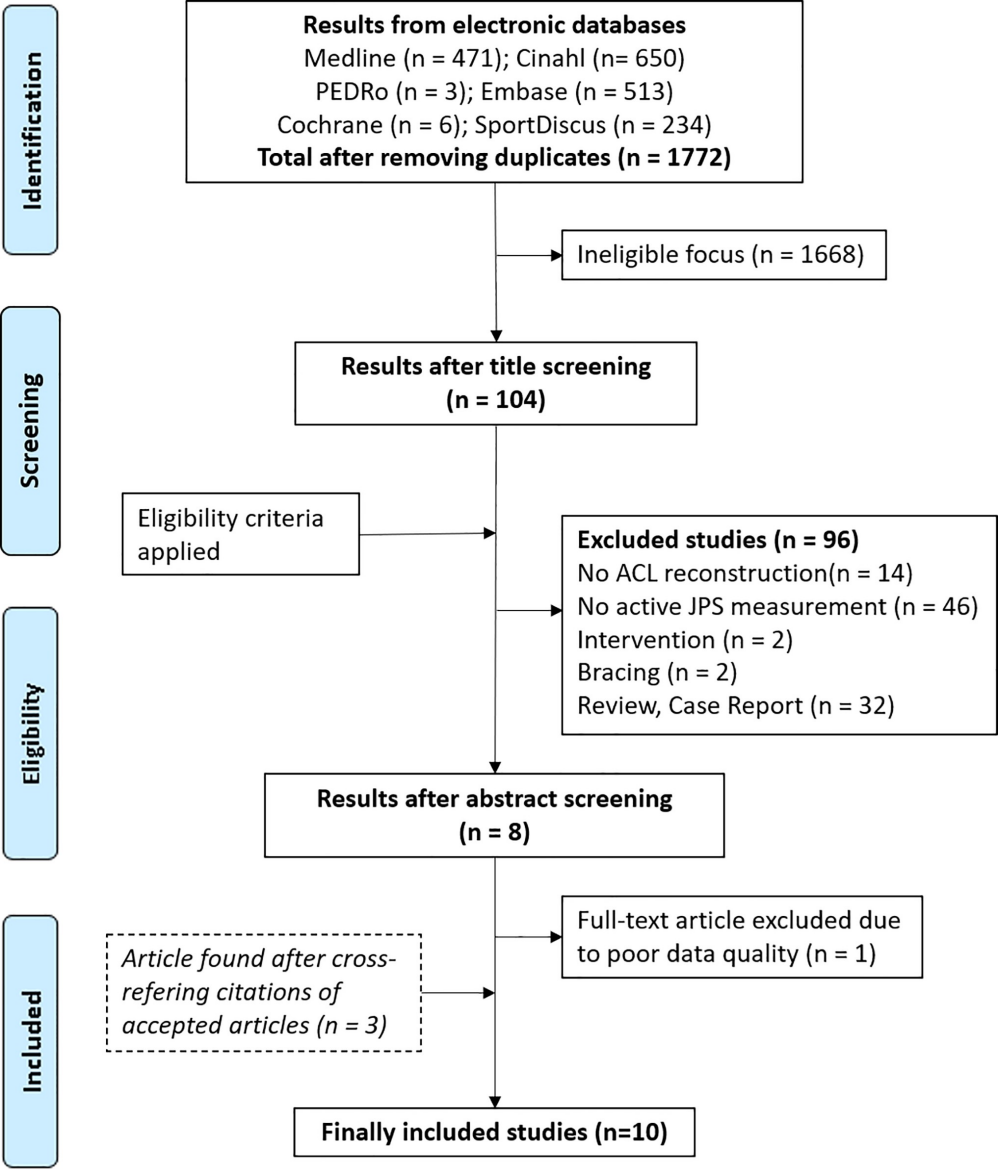
Flow diagram of the study selection process according to PRISMA [12]. Legend: ACL = Anterior cruciate ligament; JPS = joint position sense test.

Initial search identified 1877 studies in the databases. After removing 105 duplicates, 1668 studies were discarded due to ineligible focus during title screening. Abstracts of 104 studies were assessed after which 96 studies were excluded. Eight studies met the inclusion criteria and three studies were additionally included after manual search of the references of the included studies. One study [16] had to be excluded during full-text reading due to poor quality of the described results. In total, 10 studies were included after application of selective criteria. The procedure is displayed in a flow chart (Fig 1).

### Study characteristics

Eight studies used a cross-sectional [17–24] and two a prospective study design [7, 25]. Participant characteristics are described in Table 1. Sample size ranged from 10 to 48 in the included studies. Participants age ranged from 16 to 54 years, and in total 265 men and 99 women were examined. The physical activity was not consistently described. Three studies reported a Tegner Score [20, 21, 24] and one stated that the participant groups had the same Tegner Score without the actual number [23]. Two studies included elite athletes [17, 18] and one study nonprofessional athletes [25]. Three studies did not reported the activity level of the participants [7, 19, 22]. The most reported surgery type was bone patellar tendon bone autograft [7, 18, 20–22, 25], followed by hamstring tendon autograft [7, 18, 23–25]. Further, it should be noted that different surgery types in the same or in multiple study populations were reported. Time between surgery and study participation varied among the included studies with a range from 3 weeks to 8 years. Three studies excluded associated injuries within the study population [17, 24, 25], while four studies did not described additional injuries of the included participants [18, 19, 21, 22] (cf. Table 1).

**Table 1:** Participant characteristics of the included studies.

Author (year)	Participant Characteristics						
	*Participants*	*Male / Female*	*Age (M±SD) in years*	*Physical activity*	*Surgery type*	*Time between surgery and study*	*Associated injuries*
*Anders et al*. *(2008)*	45 ACL-R	35/10	31 (range 19–51)	NM	BPTB autograft	average 36 months	NM
*Angoules et al*. *(2011)*	20 ACL-R (HTA) 20 ACL-R (BPB)	16/4 18/2	31 (range 17–54)	Nonprofessional athletes, no further details provided	Arthroscopic ipsilateral HTA or BPTB autograft	3, 6, 12 months	No associated injuries
*Baumeister et al*. *(2008)*	10 ACL-R 12 Con	7/3 9/3	27 ± 5 25 ± 3	Recreational athletes Tegner Score pre-injury: > 5 at study: 4.5 ± 1.5	HTA	12.5 ± 4.6 months	No associated injuries
*Fischer-Rasmussen & Jensen (2000)*	18 ACL-R 20 Con	9/9 11/9	27 ± 5 27 ± 4	Tegner score (ACL-R) (workload): 3.6 ± 1.6 (athletics): 4.9 ± 2.5	BPTB autograft or STA	NM	NM
*Guney-Deniz et al*. *(2020)*	22 ACL-R (QTA) 22 ACL-R (HTA) 21 ACL-R (TAA) 20 Con	17/5 18/4 17/4 NM	27.8 ± 3.1 26.7 ± 4.6 26.4 ± 5.5 28.7 ± 3.1	Same Tegner Score but NM	QTA, HTA & TAA	13.5 ± 2.1 months 13.3 ±1.8 months 13.1 ±1.9 months	Articular cartilage defects and multiple ligament injuries excluded
*Harter et al*. *(1992)*	48 ACL-R	30/18	27.6 ± 6.9	NM	PTA or STA as intraarticular or iliotibial band tenodesis as extraarticular substitution	4.1 ± 1.7 years (range 2 to 8.4)	NM
*Moussa et al*. *(2008)*	26 ACL-R 26 Con	26/0 26/0	22 ± 3.1 22.8 ± 1.6	Elite athletes (National A Soccer)	PTA	24 months (± 1 week)	No associated injuries
*Ozenci et al*. *(2007)*	20 ACL-R (Autograft) 20 ACL-R (Allograft) 20 Con	20/0 16/4 17/3	29.5 ± 6.9 30.2 ± 4.6 27.6 ± 2.6	Tegner score (ACL-R) preinjury: 7.5 ± 0.89 7.0 ± 1.45 postinjury: 3.6 ± 1.4 2.7 ± 1.26	BPTB autograft BPTB allograft	16.5 ± 5.5 months 25.6 ± 13 months	Meniscal injury (other additional injuries excluded)
*Reider et al*. *(2003)*	26 ACL-R 26 Con	15/11 13/13	25 (range 16–48) 25 (range 18–40)	NM	HTA and BPTB autograft	3, 6 weeks and 3, 6 months post Op	Medial and lateral meniscal injury
*Relph and Herrington (2016)*	10 ACL-R 10 Con	3/7 3/7	22.4 ± 3.75 22.1 ± 4.07	Elite athletes Con (healthy; activity, age, gender, sport matched)	6x HTA, 4x BPTB autograft	17.9 ± 4.68 months	NM

ACL-R = ACL reconstructed; Con = Control; HTA = Hamstring tendon autograft; BPTB = Bone patella tendon bone; STA = Semitendinosus tendon autograft; QTA = Quadriceps tendon autograft; TAA = Tibialis anterior allograft; PTA = Patellar tendon autograft; NM = Not mentioned; M = Mean; SD = Standard Deviation.

### JPS testing

The JPS test design and results of all included studies are presented in Table 2. The starting angle of the JPS test was mostly full knee extension (0°) [7, 17, 18, 20, 21, 23, 25] or 90° knee flexion [18, 19, 24]. One study reported a second starting angle of 60° knee flexion and another study let the angles be self-selected by the participants [21, 22]. Most studies used defined target angles ranging from 15° to 75° flexion or extension. However, three studies chose random target angles [7, 20, 22]. Two studies reported two practice trials prior to the testing [7, 20] and one study let the participants familiarize themselves with visual feedback during five movements before each measurement trial [24]. A self-selected movement velocity was described in two studies [23, 24], while the rest did not provide details about the movement velocity. Further, one study reported which leg was first examined (if applicable) during the testing [24]. Five studies compared the affected limb with the contralateral side and to a control group [7, 17, 18, 20, 21]. Three studies compared to the contralateral side [19, 22, 25] and two compared to a control group [23, 24].

**Table 2:** Study design and results of the included studies.

Author (year)	Joint position sense test				
* *	*Comparison*	*Knee ROM*	*Test design*	*Measurement device*	*Results (mean ± standard deviation)*
*Anders et al*. *(2008)*	Contralateral	From and to a self- selected knee angle	Measurements in seated and supine position. Participants chose an angle and set this angle with a 5 s holding in a second attempt. Each test was repeated three times	Electrogoniometer	Sitting: Significantly higher mean variance for injured (2.78°) compared to contralateral (2.60°) limb (p = 0.012).
					Supine: No significant difference found.
*Angoules et al*. *(2011)*	Contralateral	From a starting angle of 0° (full extension) to target angle of 15°, 45° and 75° knee flexion	Participants were blindfolded and prevented from audio cues. Passive positioning then active repositioning. Three repetitions per measured angle.	Dynamometer	Significantly higher angular deviation 3 months post op in the reconstructed (recon) compared to contralateral leg in both groups at 15°: recon 3.90° ± 1.48°, contralateral 1.98° ± 0.92° (p < 0.0005); 45°: recon 3.72° ± 1.50°, contralateral 2.20° ± 0.85° (p < 0.0005) and 75°: recon 3.65° ± 1.23°, contralateral 2.52° ± 1.10° (p = 0.001)
					No significant differences 6- and 12-months post op.
*Baumeister et al*. *(2008)*	Contralateral and control	From a starting angle of 90° (0° = full extension) to target angle of 40° knee flexion	Participants seated and familiarized with visual feedback for 5 repetitions. Reproduction task without visual feedback and holding at target angle for 3 s. Repeated reproduction for 3 min per trial and 4 trials per limb.	Training machine M3 (Schnell) with electrogoniometer	Significantly larger error in the injured limb compared to Con (0.021 > p > 0.012).
					Interaction effect between the trials in ACL-R and Con (p = 0.036).
*Fischer-Rasmussen & Jensen (2000)*	Contralateral and control	From a starting angle 0° (full extension) or 60° flexion to target angle of 15°, 20°, 25°, 30° and 35° knee flexion	Participants in supine position, blindfolded and with earplugs. Passive positioning and active reproduction. Reproduction of the target angles and holding at angle for 3 s in random order. 20 trials (4 at each angle) per limb.	Custom build test bench	Significantly higher error in ACL-R (3.50° ± 1.37°) compared to Con (2.70° ± 1.42°) when starting position was 60° flexion (p = 0.01) but not with starting from full extension.
*Guney-Deniz et al*. *(2020)*	Control	From a starting angle 0° (full extension) to target angle of 15°, 45° and 75° knee flexion	Participants seated and blindfolded. Self-selected movement velocity and holding at target angle for minimum of 10 s. 6 repetitions per angle.	Dynamometer	Significantly higher error in ACL-R groups (HTA: 2.7°; TAA: 3.2°; QTA: 2.3°) compared to Con (0.4°) at 15° (p < 0.001).
					No significant differences at other target angles.
*Harter et al*. *(1992)*	Contralateral	From a starting angle of 90° (0° = full extension) to target angle of 15°, 20°, 25°, 30° and 35° knee flexion	Participants seated and blindfolded. Passive positioning (approx. 10–15°/s and held at target angle for 3 s) then active reproduction of the target angle in random order and with verbal notice if reached.	Dynamometer	No significant difference between the reconstructed and contralateral limb and between the surgery types at any target angle
*Moussa et al*.* (2008)*	Contralateral and control	From a starting angle of 0° (full extension) to target angle of 15° and 60°	Participants seated and blindfolded. Passive positioning of the uninjured or dominant knee/limb then active reproduction of the injured/non dominant knee/limb. 3 repetitions per angle	Electrogoniometer	Significantly higher error at 15° in the reconstructed (5.5° ± 0.1) compared to contralateral (3.6° ± 1.5°) limb (p < 0.05) and healthy control group (p = 0.04).
					No significant difference at 60° target angle
*Ozenci et al*. *(2007)*	Contralateral and control	From a starting angle of 0° (full extension) to a randomly selected target angle	Participants seated, blindfolded and prevented from audio cues. Passive positioning then active reproduction. 10 repetitions of randomly predetermined positions for each leg.	Dynamometer	No significant differences between the groups.
*Reider et al*. *(2003)*	Contralateral and Control	From a starting angle of 0° (full extension) to a randomly selected target angle	Participants seated, blindfolded and prevented from audio cues. Passive positioning then active reproduction. 10 repetitions of randomly predetermined positions for each leg.	Electrogoniometer	Significantly less error in reconstructed (5.67°) compared to contralateral (6.91°) (p = 0.011). limb and control (7.53°) (p = 0.008) at 6 months post op. No significant differences were found at the other time points.
*Relph and Herrington (2016)*	Contralateral and control	From a starting angle of 0° (full extension) to a target angle from 30–60° and from a starting angle of 90° to a target angle from 60° - 30°	Participants seated and blindfolded. Passive positioning (approx. 10°/s and held at target angle for 5 s) then active reproduction. 5 trials per target angle and both legs of ACL-R and dominant leg of Con.	Camera (Image capture technique)	Significantly higher error in reconstructed knees (8.1° ± 1.24°) compared to contralateral limb (3.5° ± 0.72°) and healthy Con (3.1° ± 1.84°) during flexion (p = 0.0001) and extension (ACL-R: 7.2° ± 0.97°; contralateral: 1.9° ± 0.47°; Con: 2.8 ± 1.94°, p = 0.0001).

ROM = Range of motion; ACL-R = ACL reconstructed; Con = Control; HTA = Hamstring tendon autograft; QTA = Quadriceps tendon autograft; TAA = Tibialis anterior allograft; op = operation.

Overall two studies revealed no significant difference in the performance of the JPS test comparing the affected limb with the contralateral side and control group [19, 20] and one study showed significantly less error in the ACL-R limb compared to a healthy control group 6 months post-operative [7]. The other included studies found significant higher error rates during the JPS test or during reproduction of some of the assessed angles in the reconstructed leg compared to either the contralateral side, control group or both [7, 17, 18, 21–25] (cf. Table 2).

A division in subgroups according to the time interval between surgery and study showed significantly higher angular deviation in the ACL reconstructed limb compared to the contralateral leg (p < 0.001) three months post-surgery but no significant deviation after 6 months [25]. Another study found higher deviations reaching no significant difference at three and six weeks and 3 months post-surgery (0.111 > p > 0.745) and a significantly better JPS 6 months after surgery in the injured leg compared to the contralateral side (p = 0.011) [7]. Studies evaluating the JPS performance approximately 12 months after surgery showed inconsistent results. One study found no significant differences comparing both limbs of the ACL-R group [25]. A significantly (p < 0.05) higher error was shown in the ACL injured limb compared to controls by another study [24]. While a third study reported a significantly higher error at a target angle of 15° knee flexion (0° = full extension) (p < 0.001) but not at the other target angles of 45° and 75° knee flexion in the ACL reconstructed limb compared to controls [23]. JPS testing more than one year after ACL reconstruction was evaluated in five studies with a range from 16 months to 8 years. Three studies found no significant difference in the injured limb compared to the contralateral side and/or control group [19, 20, 22]. Two studies showed significantly higher error rates in the ACL reconstructed limb at a target angle of 15° (0° = full extension) (p < 0.05) and during flexion and extension (p < 0.0001) compared to the contralateral side and healthy control group [17, 18].

### Methodological quality

The quality assessment yielded an overall fair quality of the included studies (cf. Table 3). Two studies were rated of poor [19, 21] and three of good quality [7, 18, 25]. The largest deficit in quality was found in the items (5) “Was a sample size justification, power description or variance and effect estimates provided?” (8/10 studies [7, 17–25]), (11) “Were the outcome measures (dependent variables) clearly defined, valid, reliable, and implemented consistently across all study participants?” (6/10 studies [7, 17, 19–22, 24]), (12) “Were the outcome assessors blinded to the exposure status of participants?” (8/10 studies [17–24]) and (14) “Were key potential confounding variables measured and adjusted statistically for their impact on the relationship between exposure(s) and outcome(s)?” (10/10 studies [7, 17–25]).

**Table 3:** Methodological quality of included studies.

Author (Year)	(1)	(2)	(3)	(4)	(5)	(6)	(7)	(8)	(9)	(10)	(11)	(12)	(13)	(14)	Rating
*Anders et al*. *(2008)*	No	Yes	Yes	Yes	No	Yes	Yes	No	No	NA	No	No	NA	No	Fair
*Angoules et al*. *(2011)*	Yes	Yes	Yes	Yes	No	Yes	Yes	Yes	Yes	NA	Yes	Yes	NM	No	Good
*Baumeister et al*. *(2008)*	Yes	Yes	Yes	Yes	No	Yes	Yes	No	Yes	NA	No	No	NA	No	Fair
*Fischer-Rasmussen & Jensen (2000)*	Yes	Yes	Yes	No	No	Yes	NM	NM	No	NA	No	No	NA	No	Poor
*Guney-Deniz et al*. *(2020)*	Yes	Yes	Yes	Yes	No	Yes	Yes	Yes	Yes	NA	Yes	No	NA	No	Fair
*Harter et al*. *(1992)*	Yes	Yes	Yes	No	No	Yes	Yes	No	No	NA	No	No	NA	No	Poor
*Moussa et al*. *(2008)*	Yes	Yes	Yes	Yes	No	Yes	Yes	No	Yes	NA	Yes	No	NA	No	Fair
*Ozenci et al*. *(2007)*	Yes	Yes	Yes	Yes	No	Yes	Yes	Yes	Yes	NA	No	No	NA	No	Fair
*Reider et al*. *(2003)*	Yes	Yes	Yes	Yes	Yes	Yes	Yes	No	Yes	NA	No	Yes	Yes	No	Good
*Relph and Herrington (2016)*	Yes	Yes	Yes	Yes	Yes	Yes	Yes	No	No	NA	Yes	No	NA	No	Good

Detailed explanation of the items (1)–(14) can be found in the quality assessment of included studies.

NA = not applicable; NM = not mentioned.

## Discussion

The results of this systematic review indicated an altered sensorimotor function after ACL reconstruction compared to the contralateral leg and to a healthy control group measured during an active knee JPS test. Although, most studies reported significantly higher error rates in the ACL reconstructed leg, some also showed no significant differences [19, 20] or even a better accuracy compared to the contralateral side and/or a healthy control group [7].

Two prospective studies in this review evaluated the JPS during the early phase of rehabilitation after ACL reconstruction. They found a higher angular deviation in the affected limb compared to the contralateral side and control, reaching statistical significance in one study, in the first weeks and three months after reconstruction [7, 25]. Six months after reconstruction there were either no significant differences [25] or significantly less error in the ACL reconstructed limb compared to the control group but no significant differences compared to the contralateral side [7] reported. Another study compared pre-operative deviations to post-operative in the affected limb. They showed no significant changes in the ACL injured limb in the timeframe before surgery and three to six months post-operative, and significantly less error nine to 24 months post-operative [26]. The described studies indicated less accurate sensorimotor function early after reconstruction. Less error in the JPS test after six months might be the result of wound healing and rehabilitative processes [25]. However, included studies examining the JPS at time points one year or more after reconstruction showed inconsistent findings. Thus, changes might not only be due to time after injury and/or treatment [5], making it difficult to draw comparisons and conclusions from them.

Potential confounding variables are discussed in the following section. The age range of the included participants was wide but could be summarized as a group of young adults. Differences in sensorimotor performances have been seen comparing young and old participants [14]. Therefore, an age effect between the studies can be disregarded. Sex differences are thought to have an influence on knee proprioception [27, 28]. Participants of both sexes were mostly examined in the studies of the present review. This might lead to false outcomes and would need a separate evaluation to exclude possible misinterpretation. Additionally, the present review showed that females are underrepresented, demanding more balanced participant recruitment. Furthermore, it is discussed that physical activity has an effect on joint proprioception, although, the entire understanding of the mechanism still needs to be investigated [14]. The reviewed studies inconsistently reported the physical activity of the ACL-R group as well as the (matched) control group, making an intra-group and inter-study comparison difficult. Possible associated injuries as well as intra- or post-operative complications are present [29] and might affect the sensorimotor function in different ways. The current review showed that four out of ten studies did not report associated injuries [18, 19, 21, 22]. This additional information might be valuable in the participant characterisation. Nevertheless, various options of associated injuries and complications are making a comprehensive differentiation difficult. Another confounding variable could be the classification of persons after ACL injury as coper and non-coper, determined by the functioning level of the knee [30]. Literature has shown that there was no proprioceptive difference between defined ACL deficient coper and non-copers [30]. One study included ACL reconstructed athletes after return to competition and reported a proprioceptive deficit compared to a control group [18]. Thus, alterations in proprioception might be equally present in well and poor functioning persons after ACL injury and reconstruction but this aspect needs further evaluation.

Overall, poorer sensorimotor function presented in this review confirms results of previous conducted meta-analysis [10, 11]. A pooled mean difference of 1.25° (95% CI, 0.72°-1.78°; p < 0.001) in the active JPS test comparing ACL deficient legs with the contralateral side was reported [10]. Another meta-analysis showed a pooled standard mean difference of 0.52° mean angle error (95% CI, 0.41 to 0.63; p < 0.001) comparing the affected to the contralateral limb and 0.35° (95% CI, 0.14 to 0.55; p = 0.001) comparing the injured leg to a healthy control group was reported [11]. However, it needs to be noted that this analysis included both, ACL deficient and reconstructed participants and presented the combined results of active and passive JPS testing [11]. The aforementioned angular deviations are significant but rather small. A limb difference of 0.1° in a healthy control group has been described and argued that differences within ACL patient groups or compared to external controls are unlikely to be clinically or functionally important and might be classified as measurement error [4]. Recently, studies consistently described that clinically important differences during a JPS test need to be at least 5° angular error [10, 18]. This must be considered when evaluating results of studies examining the sensorimotor abilities of ACL injured persons. Moreover, due to varied testing modalities, different receptors are addressed [31]. During active JPS tests leg muscles are actively contracted. It is assumed that muscle spindles are a main source of proprioceptive information, however discussion on contributions of other receptors and their correlation during functional and full-range joint movement is ongoing [32, 33]. Therefore, comparisons of the present review to other studies examining sensorimotor performance with e.g. passive JPS or threshold to detect passive motion methods need to be carefully evaluated.

Inconsistencies in the results of the included studies, e.g. with significant differences examined at one target angle but not at others, were present [7, 17, 23]. A possible factor might be the JPS assessment technique used. Literature examining the reliability of JPS testing showed moderate to good but variable intra-rater reliability and good inter-rater reliability [8]. Thus, differences within a study might be due to reliability deficits. Moreover, trial numbers might be too small to detect differences or deficits in the error rates. It is discussed that at least 10 repetitions are necessary to diminish the insufficient accuracy in proprioceptive performance [31]. Often, these trial numbers were not reached in the included studies. Additionally, only three studies reported a sample size, post-hoc power analysis and/or reliability measures [7, 18, 25]. Accordingly, future studies should implement e.g. sensitivity and reliability tests [11] to ensure good methodological quality. Based on the large heterogeneity in the designs of the included studies and the limiting factors described, a meta-analysis was dismissed.

The clinical and functional relevance of the JPS test has been questioned [4]. Nevertheless, the JPS test can be performed in a non-weight bearing and controlled situation. It gives the opportunity to measure the sensorimotor performance in the acute injury phase, early after the ACL rupture or reconstruction. This might give valuable insights into the sensorimotor function during early processes of rehabilitation and recovery after ACL injury. In addition, active test protocol causes stimulation of joint and muscle receptors, reflecting a more functional assessment compared to the passive procedure [34]. Further, neuromuscular training could be extended by the active or passive joint position sense test. This might be a useful treatment especially in the early rehabilitation phase, as described above.

Some of the included studies have shown sensorimotor deficits in both limbs of the ACL-R group [19–21, 25], confirming findings in the literature [4, 35]. It is argued that alterations in intra- and periarticular receptors of the affected leg also influence the contralateral side due to central nervous system (CNS) modifications [24, 36]. To fully understand the components of human movement, studies focusing on CNS processes during sensorimotor tasks were initialized in the past [31]. However, peripheral and central mechanisms underlying the sensorimotor control are still unclear demanding further research [24].

There are some study limitations. The results are limited to the sensorimotor performance in ACL reconstructed participants during an active JPS task. This needs to be considered when comparing this research to other reviews using a different method to measure proprioception or sensorimotor function. Further, errors or deviations between reproduced and targeted angle were not entirely and specific reported in some studies [17, 21, 24]. Thus, evaluation of the clinical relevance is difficult and reflects the need of precise and ample results description. The quality assessment tool for observational and cross-sectional studies provides detailed quality evaluation but lacks a scoring system. The aim was to evaluate the sensorimotor function assessed with the active JPS test and was not intervention or exposure driven. Missing of an overall score limits the comparability between the studies but characteristics and content of the assessment tool outweigh this disadvantage. None of the studies reported pre-injury sensorimotor performance. A possible predisposition of altered sensorimotor function may be a factor for a later ACL rupture [37]. Differences might not be the cause of the rupture and reconstruction.

## Conclusion

Knee sensorimotor function is affected in an ACL ruptured and reconstructed limb determined in an active JPS test compared to the contralateral leg and healthy control group. Results of this systematic review may indicate a poorer sensorimotor performance; however, the small magnitude may suggest little effect on the clinical relevance. Various measurement methods and mostly cross-sectional designs with a great range of time between injury and investigation make comparison of the studies with each other difficult. Furthermore, prospective studies, during the early injury phase, can be conducted with active JPS testing giving valuable insights into sensorimotor function during rehabilitative process.

## Supporting information

S1 Checklist PRISMA checklist. (PDF)

S1 File Search strategy. (PDF)
