# Supplementary material for: Alterations in sensorimotor function after ACL reconstruction during active joint position sense testing. A systematic review
Source: PLoS One. 2021 Jun 25;16(6):e0253503. doi: 10.1371/journal.pone.0253503 (PMC8232438; doi:10.1371/journal.pone.0253503)
Supplement: S1 File — (PDF) [file pone.0253503.s002.pdf]

S1 Search strategy.

Database	Search terms	Filter options
MEDLINE	((((((Anterior cruciate ligament) OR (ACL)) AND (reconstruction)) OR (surgery)) OR (repair)) AND (joint position sense test)) OR (JPS test)	Humans
CINAHL	TI anterior cruciate ligament OR acl AND TI reconstruction OR repair OR surgery AND TI joint position sense test OR JPS test	Research Articles, Exclude MEDLINE records, Human
EMBASE	(('anterior cruciate ligament': ti,ab,kw OR acl:ti,ab,kw) AND reconstruction:ti,ab,kw OR surgery:ti,ab,kw OR 'repair'/exp OR repair) AND ('joint position sense test' OR (('joint'/exp OR joint) AND ('position'/exp OR position) AND ('sense'/exp OR sense) AND ('test'/exp OR test))) OR 'jps test' OR (jps AND ('test'/exp OR test))	Human, Publication types: Article, Article in Press
PEDro	Abstract & Title (Anterior cruciate ligament OR ACL) AND (reconstruction OR surgery OR repair) AND (joint position sense test OR JPS test)	Body part: Lower leg or knee
Chochrane Library	(TAK) Anterior cruciate ligament OR ACL AND (TAK) reconstruction OR repair OR surgery AND (TAK) joint position sense test OR JPS test	
SPORTDiscus	TI anterior cruciate ligament OR acl AND TI reconstruction OR repair OR surgery AND TI joint position sense test OR JPS test	Human
